# Analysis of vancomycin use and associated risk factors in a university teaching hospital: a prospective cohort study

**DOI:** 10.1186/1471-2334-7-88

**Published:** 2007-08-01

**Authors:** Moacyr S Junior, Luci Correa, Alexandre R Marra, Luis FA Camargo, Carlos AP Pereira

**Affiliations:** 1 Department of Infectious Disease, Universidade Federal de Sao Paulo, Sao Paulo, Brazil

## Abstract

**Background:**

Vancomycin use is considered inappropriate in most hospitals. A particular concern is the recent emergence of *S. aureus *with decreased susceptibility to vancomycin, making it important to reduce overall exposure to vancomycin to minimize the incidence of VRE (vancomycin-resistant enterococci). The aim of this work was to analyze the use of vancomycin and the risk factors associated with inappropriate treatment.

**Methods:**

A prospective survey was conducted on all patients receiving vancomycin between 1^st ^March 2002 and 30^th ^September 2002 in a university-school hospital. Appropriateness of vancomycin use was assessed, according to the criteria established by the Centers for Disease Control and Prevention (CDC), at two time points: first, at the beginning of therapy, and second, continuing after 72 hours.

**Results:**

A total of 557 patients received vancomycin. Three hundred seventy-four (67.1%) were under 60 years old, 374 (67.1%) had prolonged stays (>two weeks) in hospital, and 455 (81.7%) were in the intensive care unit (ICU). Two hundred sixty-three patients (47.2%) had some invasive device. In 324 (58.2%) patients the duration of vancomycin treatment was up to two weeks. Vancomycin was inappropriately used in 65.7% during the first 24 hours and in 67% at the 72 hours point according to CDC criteria [4]. The inappropriateness of vancomycin use during the first 24 hours was related to: patients aged less than 60 (OR 1.7; CI 95% 1.1–2.5), non-ICU patients (OR 1.5; CI 95% 1.0–2.4) and patients without neutropenia (OR 7.5; CI 95% 2.4–22.7). At 72 hours, the inappropriateness of vancomycin use was related to: patients aged less than 60 (OR 1.5; CI 95% 1.0–2.3), non-ICU patients (OR 1.7; CI 95% 1.1–2.7) and patients without neutropenia (OR 8.0; CI 95% 2.6–24.3).

**Conclusion:**

Vancomycin was abused. Patients aged less than 60, non-ICU patients and those who did not present neutropenia were the principal groups at risk of inappropriate use.

## Background

In recent decades, increases have been observed in antibiotic consumption and resistance to antimicrobials, mainly in large hospitals [1]. More than half of all patients admitted receive antibiotics. It is believed that 25–50% of all prescribed antibiotics are inappropriate in respect of drug choice, dose administered or duration of treatment [2].

The development of vancomycin resistance illustrates the alternation of success and failure that has characterized the history of the antimicrobial age. This age started with penicillin, the discovery of which was revolutionary. Later, vancomycin proved active against all Gram-positive cocci and for a time no resistance was found. It has become one of the most frequently prescribed antibiotics in hospitals, especially because of the increase of infections caused by methicillin-resistant *Staphylococcus aureus *(MRSA) and *Staphylococcus *coagulase-negative [2]. Abuse of this drug has favored the emergence of vancomycin-resistant *Enterococcus *sp [3,4].

With the aim of reducing vancomycin resistance, the CDC produced directives to orientate the use of this antibiotic. Despite this initiative, several studies have shown that the use of vancomycin remains high and, in about 34–67% of cases, is considered inappropriate [1]. This inappropriate usage may be due to the high level of resistance present in hospitals where the MRSA and methicillin-resistant *Staphylococcus *coagulase-negative are endemic[6].

The objective of this study was to evaluate the appropriateness of vancomycin use, according to the rules of the Hospital Infection Control Practices Advisory Committee (HICPAC) [4], in a university-school hospital that has high incidences of resistant *Staphylococcus aureus *and methicillin-resistant *Staphylococcus *coagulase-negative (46.4% and 42.2%, respectively). We also aimed to identify the risk factors associated with its inappropriate use.

## Methods

### Setting

The study was carried out in the Federal University of Sao Paulo a teaching hospital between March and September 2002. It had previously been approved by the Ethics in Research Committee. This 644-bed hospital performs kidney, heart, bone marrow, liver and pancreas transplants.

As vancomycin is a restricted-use antibiotic in the hospital, prescription depended upon evaluation by the Antimicrobial Rationalization Service. This department evaluates all requests for restricted-use antimicrobials according to completed requirement forms. When the infectious disease specialist staff informed the assistant physician about partial Gram-positive blood culture results and, subsequently, the final results with complete identification of the respective antibiogram, the data and recommended antimicrobial therapy were discussed and alterations could be suggested. One of the service infectious disease specialists would make daily evaluations of patients who had used vancomycin until discharge or death, and would assess the indication for vancomycin according to the HICPAC criteria [4].

The vancomycin prescription was prescribed by medical residents in their respective specialty. They were oriented by staff physicians, whose average years of qualification were five years.

### Study Design

To evaluate the appropriateness of vancomycin use, a prospective cohort study was performed on patients who had used the drug. Appropriateness was judged according to the CDC criteria (Table 1) [4]. All patients who had been treated more than once with vancomycin or who had been treated for less than 24 hours were excluded from the study.

**Table 1:** Criteria for appropriate vancomycin use according to the CDC criteria [4]

**Appropriate use**
Serious infections caused by b-lactam-resistant Gram-positive microorganisms;
Infection caused by Gram-positive organisms in patients allergic to b-lactam antimicrobials;
Antibiotics treatment for colitis when there is a problem with metronidazole use or imminent life risk;
Surgical prophylaxis, with prosthesis implant, in institutions with high rates of oxacillin-resistant Gram-positive infections;
Neutropenics with extensive mucosite, infection related to venous catheters, previous prophylaxis with fluorquinolone, hypotension or sepsis.
**Inappropriate use:**
Routine surgical prophylaxis;
Febrile neutropenia that does not present isolation of oxacillin-resistant Gram-positive bacteria;
Treatment of a single blood culture for oxacillin-resistant Staphylococcus, coagulase-negative, if another culture collected simultaneously was sterile;
Empirical use, continuous, in patients whose cultures are negative for Gram-positive bacteria;
Presence of catheter and fever;
Decontamination of the gastrointestinal tract;
Prophylaxis for low birth weight infants;
Primary treatment of colitis by antibiotics;
Colonization by oxacillin-resistant Gram-positive bacteria;
Prophylaxis for patients in continuous peritoneal dialysis or hemodialysis;
Convenience treatment of infections by b-lactam-sensitive Gram-positive in hemodialysis patients;
Topical vancomycin use.

The indication was assessed at the first 24 hours and after 72 hours, according to the CDC criteria [4]. These criteria are described in Table 1, which relates to vancomycin treatment of patients with (1) infections caused by β-lactam-resistant Gram-positive microorganisms, (2) allergies to this same class of antibiotics or (3) failure to respond to metronidazole therapy for pseudomembranous colitis.

The patients were evaluated according to their clinical and demographic characteristics. The variables analyzed were: sex, age, length of stay, ward, admission diagnosis, according to ICD-9-CM (International Classification of Diseases, nine revision, clinical modification), disease classification according to McCabe-Jackson criteria [7], prior surgery, presence of neutropenia, mechanical ventilation, presence of a central venous catheter, and period and outcome of vancomycin use.

### Statistical Analysis

The obtained data from the completed forms were stored in an ACCESS database, version 2.0, Microsoft C.O., Ireland. Univariate analysis of the risk factors was done by Chi-square (X^2^) or Fisher's Exact Tests and continuous variables were analyzed by Student's t test. A multiple logistic regression model was used for multivariate analysis. *P *values lower than 0.05 were considered statistically significant. The OR (odds ratio) and its respective confidence interval estimates were 95%. Statistical analyses were performed using SAS software version 8.0 (Statistical Analysis System, Cary, NC, USA).

## Results

During the study period, 667 treatments were evaluated. Only patients who had used vancomycin a single time were analyzed (557; 83.5%).

The average length of hospital stay was 39.8 days; the average length of hospital stay prior to vancomycin use was 15.1 days. The average total time of vancomycin use was 14.3 days, representing the majority of patients (324, 58.2%) in our analysis. The clinical and demographic characteristics of the patients are listed in Table 2. Of the patients studied, the average age was 49 years, 56.6% were men, 47.2% were hospitalized in the ICU, Pediatrics and Adult. Two hundred thirty six was submitted surgery included gastrointestinal surgery in (194, 82.2%) patients, genitourinary in (14, 6%) patients, neurological surgery in (12, 5%) patients, orthopedic surgery in (8, 3.4%) patients and others surgery in (8, 3.4%) patients. Central venous catheter was present in (354, 63.6%) patients. Most (81.7%) patients had been hospitalized for at least two weeks, and the principal reason for hospitalization was infectious disease (22.6%). According to the McCabe-Jackson criteria, 72% of the patients had potentially fatal diseases; by the criteria of Charlson, 87% of the patients   had score under of 4  [8]. The general mortality rate was 47%.

**Table 2:** Demographic data of the 557 Sao Paulo Hospital patients who used vancomycin between March 1^st ^and September 30^th ^2002

**Variables**	**N**	**Percentage**
**Sex**		
Male	315	56.6
Female	242	43.4
**Age**		
< 60 years	374	67.1
≥ 60 years	183	32.9
**Length of hospital stay**		
< 2 weeks	102	18.3
≥ 2 weeks	455	81.7
**Unit**		
ICU (Pediatrics and general)	263	47.2
General practice	158	28.4
Pediatrics	71	12.7
Surgery	47	8.4
Others	18	3.2
**Diagnosis**		
Infectious diseases	126	22.6
Hematological diseases	66	11.8
Cardiac diseases	68	12.2
Malignancies disease	59	10.6
Neurological diseases	55	9.9
Gastrointestinal diseases	52	9.3
Gynecological-urinary diseases	43	7.7
Others	88	15.9
**Neutropenia**		
Yes	28	5.0
No	529	95.0
**Oral-Tracheal intubation**		
Yes	308	55.3
No	249	44.7
**Central venous catheter**		
Yes	354	63.6
No	203	36.4
**Vancomycin use period**		
< 2 weeks	324	58.2
≥ 2 two weeks	233	41.8
**Outcome**		
Discharge	295	53.0
Death	262	47.0

Vancomycin use was considered appropriate in 191 cases (34.3%) during the first 24 hours and in 184 cases (33.0%) after 72 hours. The principal indication for appropriate use was the isolation of β-lactam-resistant Gram-positive bacteria at both time points, as shown in Table 3. Correspondingly, the rates of inappropriate use were 65.7% and 67% at the 24-hour and the 72-hour, respectively, as shown in Table 4. Half the inappropriate vancomycin use (285 cases, 51.3%) at both time points was because the patients were in critical clinical conditions.

**Table 3:** Analysis of the frequency of with which vancomycin use met appropriateness criteria in Sao Paulo Hospital between March 1^st ^and September 30^th ^2002, according to the CDC criteria (HICPAC,1995), at the 24 hour and 72 hour time points

**Criteria**	**24 hours**		**72 hours**	
	
	**N**	**%**	**N**	**%**
Isolation of β-lactam-resistant Gram-positive bacteria	165	29.6	159	28.5
Presence of extensive mucositis in neutropenic patients	1	0.2	1	0.2
Infection related to catheter in neutropenic patients	1	0.2	1	0.2
Hypotension and sepsis in neutropenic patients	24	4.3	23	4.1

Total	191	34.3	184	33.0

**Table 4:** Analysis of the frequency of with which vancomycin use failed to meet appropriateness criteria in 557 patients in Sao Paulo Hospital between March 1^st ^and September 30^th ^2002, according to the CDC criteria (HICPAC,1995), at the 24 hour and 72 hour time points

**Criteria**	**At the first 24 hours**		**At the first 72 hours**	
	
	N	%	N	%
Surgical prophylaxis	6	1.1	6	1.1
Suspect of hospital infection without cultures being obtained	7	1.3	7	1.3
Critical clinical condition	285	51.3	285	51.3
Presence of catheter and fever	38	6.8	38	6.8
Decontamination of the gastrointestinal tract	3	0.5	3	0.5
Eradication of MRSA colonization	2	0.3	1	0.2
Treatment (chosen for dosing convenience) for β-lactam-sensitive Gram-positive bacteria	25	4.5	33	5.8

Total	366	65.7	373	67.0

Univariate analysis showed that the risk factors associated with inappropriate vancomycin use at the two time points, according to the CDC criteria, were patients under 60 years old, those who were hospitalized in units other than ICUs, and those without central venous catheters or with a central venous catheter for no longer than two weeks. In our analysis, patients who underwent surgery did not have risk factors associated with misuse vancomycin (Table 5). When the two time periods were examined by multivariate analysis, inappropriate vancomycin use was found to be in patients under 60 years old, without neutropenia and outside ICUs (Table 6).

**Table 5:** Univariate analyses of risk factors for inappropriate vancomycin use, at the first 24 and 72 hours, according to the CDC criteria (HICPAC,1995), in 557 patients in Sao Paulo Hospital between March and September 2002

**Variables**	**Inapropriateness (24 h)**		***p***	**Inappropriateness (72 h)**		***p***
	N(366)	%		N(373)	%	
**Age**						
< 60 years	264	(70.5)	**0.001**	266	(71.3)	**0.002**
≥ 60 years	102	(56.3)		107	(58.5)	
**Unit**						
Others	210	(71.3)	**0.001**	215	(73.4)	**0.001**
UCI/UCI ped	156	(59.7)		158	(60.1)	
**Neutropenia**						
Yes	9	(32.1)	**0.001**	8	(32.1)	**0.001**
No	357	(67.6)		365	(68.9)	
**Central venous catheter**						
Yes	221	(62.7)	**0.04**	226	(64.1)	**0.049**
No	145	(71.3)		147	(72.3)	
**Central venous catheter**						
< 2 weeks	125	(69.9)	**0.003**	127	(69.5)	**0.002**
≥ 2 weeks	96	(56.7)		99	(57.9)	

**Table 6:** Multivariate analyses of risk factors for inappropriate vancomycin use, at the first 24 and 72 hours, according to the CDC criteria (HICPAC,1995), in 557 patients in Sao Paulo Hospital between March and September 2002

**Variables**	**Odds ratio**	**IC (95%)**	***p***
**CDC 24 hours**			
< 60 years	1.7	1.16 – 2.58	**0.007**
Unit (no UCI)	1.5	1.03 – 2.44	**0.035**
Without neutropenia	7.5	2.46 – 22.79	**0.001**
**CDC 72 hours**			
<60 years	1.5	1.07 – 2.34	**0.021**
Unit (no UCI)	1.7	1.15 – 2.73	**0.009**
Without neutropenia	8.0	2.64 – 24.37	**0.001**

## Discussion

The outbreak of *Enterococcus *spp resistance and highly vancomycin-resistant *Staphylococcus aureus *in 2002 in the USA limited the therapeutic options for treating Gram-positive bacterial infections [9,10]. Even after the development of strategies to hinder the dissemination of resistance by formulating guidelines for rational use of antimicrobials and, above all, appropriate vancomycin use in clinical practice, quite the opposite has been observed: bacterial resistance has been disseminated and vancomycin use remains indiscriminate [11,12].

In our hospital, as in others [13], there were high incidences of methicillin resistant *Staphylococcus aureus*, favoring the empirical use and the abuse of vancomycin. Thus, the factors most frequently associated with inappropriate use of vancomycin were its empirical use without evidence of infection. Other authors have obtained similar results [5,14].

There was a high incidence of inappropriate vancomycin use at both time points in our study, mainly in patients who presented potentially fatal disease according to the McCabe-Jackson criteria. Even, when Gram-positive bacterial infection was not identified at the 72 hour point, therapy was not suspended and vancomycin administration was maintained because of the critical clinical condition of the patient. Take into account the high prevalence of MRSA isolate at our hospital, were considered inappropriate use in critical ill patients, also, we were not liberal in allowing its use in situation such as with presence catheter and fever because was followed CDC guidelines that is not considered adequate in these population. Although the CDC has published criteria for prudent use of vancomycin, there is no "gold standard" for determining the appropriateness of use. They are not specific, particularly with respect to MRSA endemic in the institution. Hamilton et al also observed that vancomycin use was maintained empirically despite negative cultures [15].

On the other hand, Ena et al observed that vancomycin was more frequently used in the ICU [2]. We found that inappropriate use was more frequent in patients hospitalized in other non-critical units, mostly in younger patients. These points negligence the use of vancomycin in this population supposedly not at high risk.

We found that vancomycin use was appropriate for neutropenic patients; there are certainly protocols for neutropenic patients that precisely indicate vancomycin since 1999, in our institution.

The external validity of the study was limited because this is a highly complex tertiary hospital and therefore has a higher incidence of antimicrobial resistance. Future studies must take small and medium sized hospitals or even other areas of Brazil into consideration. However, it is probably valid to extrapolate the main findings to most Brazilian tertiary and teaching hospitals: difficulty in controlling dissemination of resistance, expression of oxacillin resistance and abuse of vancomycin.

It should also be considered that the CDC criteria were not developed for hospitals with high levels of Gram-positive resistance. There is a need to develop protocols that acknowledge the real situation in each institution [16,17]. The CDC has published criteria for prudent use, but they are not specific, particularly with respect to institution with high incidence of MRSA, particularly in unstable condition even in the absence of evidence of a Gram-positive infection. Given that vancomycin use is inappropriate in younger patients (under 60 years), without neutropenia and venous catheterized for no longer than two weeks, recommendations are necessary to minimize usage in this population, since the CDC guidelines emphasize the high risk population (patients with neutropenia, prolonged catheterization and hospitalized in ICU). In view of this, it is important for future studies to highlight this subpopulation in order to minimize inappropriate use.

Thus, our study has identified the factors in vancomycin misuse and the places where use is inappropriate, making possible a more direct intervention and the elaboration of strategies to decrease this inappropriateness and that each hospital should create institution-specific guidelines for vancomycin use based on local microbial resistance patterns.

## Conclusion

Vancomycin was inappropriateness in patients aged less than 60, non-ICU patients and those who did not present neutropenia. It is necessary to elaborate in each hospital specific guidelines for vancomycin use.

## Competing interests

All the authors declare that they have no commercial association or financial involvement that might pose a conflict of interest in connection with the submitted article.

## Authors' contributions

MSJ participated in study and drafted the manuscript. LC helped to design the study and collected the data. AM performed the statistical analyses and the chart review. LFAC helped with manuscript writing and the chart review. CAPP participated in all aspects of the study including study design, provision of study patients, data analysis and interpretation. All authors participated in interpretation and drafting the manuscript, and read and approved the final manuscript.

## Pre-publication history

The pre-publication history for this paper can be accessed here:


